# DC-25 GHz and Low-Loss MEMS Thermoelectric Power Sensors with Floating Thermal Slug and Reliable Back Cavity Based on GaAs MMIC Technology

**DOI:** 10.3390/mi9040154

**Published:** 2018-03-29

**Authors:** Zhiqiang Zhang, Yao Ma

**Affiliations:** 1 Key Laboratory of MEMS of the Ministry of Education, Southeast University, Nanjing 210096, China; 2 College of Field Battle Engineering, People's Liberation Army University of Science and Technology, Nanjing 210007, China; eemaoma@gmail.com

**Keywords:** thermoelectric power sensor, wideband, GaAs MMIC, MEMS, floating slug, back cavity, microwave measurement

## Abstract

Wideband and low-loss microwave power measurements are becoming increasingly important for microwave communication and radar systems. To achieve such a power measurement, this paper presents the design and measurement of wideband DC-25 GHz and low-loss MEMS thermoelectric power sensors with a floating thermal slug and a reliable back cavity. In the sensors, the microwave power is converted to thermovoltages via heat. The collaborative design of the thermal slug and the back cavity, i.e., two thermal flow paths, is utilized to improve the efficiency of heat transfer and to ensure reliable applications. These sensors are required to operate up to 25 GHz. In order to achieve low microwave losses at the bandwidth, the floating thermal slug is designed instead of the grounded one. The effects of the floating slug on the reflection losses are analyzed by the simulation. The fabrication of these sensors is completed by GaAs monolithic microwave integrated circuits (MMIC) and micro-electro-mechanical systems (MEMS) technology. Measured reflection losses are less than −25.6 dB up to 12 GHz and −18.6 dB up to 25 GHz. The design of the floating thermal slug reduces the losses, which is equivalent to improving the sensitivity. At 10 and 25 GHz, experiments exhibit that the sensors result in sensitivities of about 51.13 and 35.28 μV/mW for the floating slug and 81.68 and 55.20 μV/mW for the floating slug and the cavity.

## 1. Introduction

With the rapid development of multi-band microwave communication and radar systems, the wideband and low-loss power measurements become more and more important for microwave signals. In recent years, the commonly used power measurement methods include three types: diode-, thermistor-, and thermopile-based microwave power sensors [1,2]. The diode-based microwave power sensors are based on employing the square law region in the nonlinear I–V curve of diodes, where the input microwave power is proportional to the low-frequency power. Using the diode method, the peak RF power is measured. As for the diode-based sensors, they are active components and require an additional attenuator when measuring the high power of mW levels. The thermistor-based microwave power sensors convert the input microwave power into heat and result in the change in resistance of the thermistors based on Joule effect, where the thermistors are generally negative resistance temperance coefficient. Using the thermistor method, the average RF power is measured. But, as for the thermistor-based sensors, they need a feedback circuit of bridge balancer and are susceptible to the external environment. So, the two types of sensors are not suitable for broadband and low-loss applications. The thermopile-based microwave power sensors convert the microwave power into heat and finally into the thermovoltage, where the thermovoltage depends on the temperature at both ends of thermocouples, regardless of the temperature profile. As for the thermopile-based sensors, they are based on Seebeck effect and output DC voltages [3,4]. These thermoelectric power sensors become a preferred choice due to zero dc power consumption, wide operation frequency, high power handling, and high linearity. However, the disadvantage of the sensors is that their sensitivity is not high. This is mainly caused by heat losses of the substrate in the conversion process of microwave power-heat-electricity. The micro-electro-mechanical systems (MEMS) technology can reduce the heat losses through locally etching the substrate and forming the membrane structure. Thermopile-based MEMS microwave power sensors have been reported widely [5,6,7,8]. Following that, the effects of the thermopile’s optimization [9], thermoelectric modeling [10,11,12], packaging [13,14], and temperature and humidity reliability [15,16] on the thermopile-based power sensors are studied. The heat losses of the substrate are reduced and the sensitivity of the sensors is increased as the substrate underneath the thermopile is thinned. It should be noted that the power sensors with a too thin membrane will bring challenges to the reliability and packaging. In order to choose the proper configuration and size of the membrane, the thermopile-based MEMS power sensors with dual thermal flow paths are proposed, where the grounded thermal slug and the cavity are included [17]. The thermal slug and the back cavity are regarded as the dual thermal flow paths. The cavity with a thicker membrane leads to good reliability, with acceptable sensitivity. Nevertheless, the grounded slug makes part of the two load resistors short circuit, resulting into smaller resistance and higher reflection losses at high frequencies. So, they only operate below 12 GHz due to the limitation of reflection losses.

In order to solve the above problem, the design of DC-25 GHz and low-loss MEMS thermoelectric power sensors with the floating thermal slug and the reliable back cavity is proposed in this paper. The back cavity has a robust membrane, and the robust membrane contributes to the packaging of the thermoelectric power sensors [13]. Here, the thermal slug is designed to be floating to obtain low microwave losses of up to 25 GHz. The microwave power sensors with the dual thermal flow paths are optimized by the simulation. The fabrication of the power sensors is based on GaAs monolithic microwave integrated circuits (MMIC) technology. Experiments demonstrate that these sensors can operate at the wide frequency range, with zero DC power consumption and low reflection losses. The design of the floating thermal slug reduces the microwave losses, which is equivalent to an improvement in sensitivity. Furthermore, the measured performances of the sensors with the floating slug are compared to the basic sensor and the reported sensors with the grounded slug. The main purpose of this work is to achieve the wideband and low-loss measurement for the sensors with dual thermal flow paths.

## 2. Structure and Design

**Figure 1:**
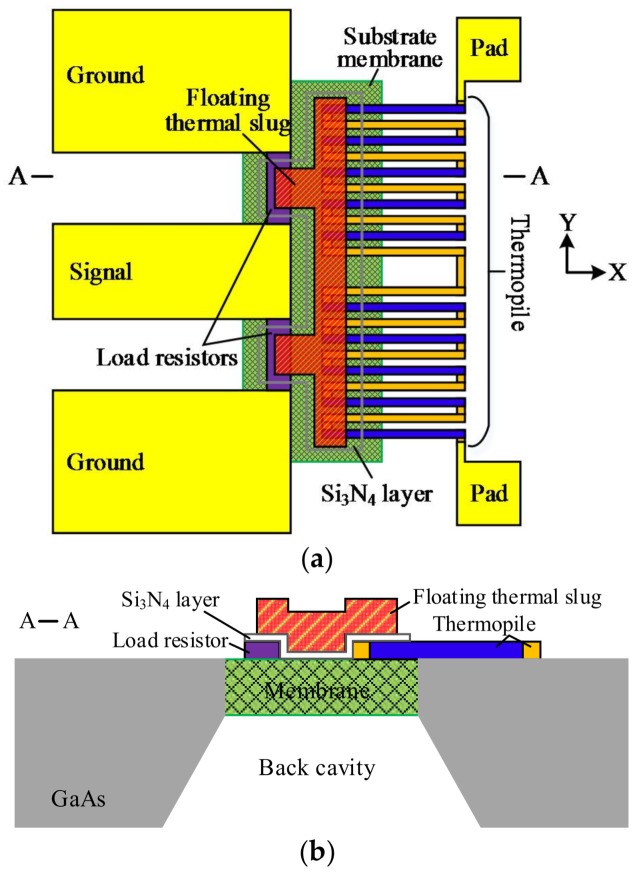
Schematic diagrams of the proposed micro-electro-mechanical systems (MEMS) thermoelectric microwave power sensor with dual thermal flow paths (floating thermal slug and back cavity) in the GaAs monolithic microwave integrated circuits (MMIC) process. (**a**) Top view; (**b**) Cross-sectional view.

Figure 1 shows the wideband and low-loss MEMS microwave power sensor with the dual thermal flow paths. It includes a coplanar waveguide (CPW) line, two load matching resistors, a thermopile, a floating thermal slug, a back cavity, and two pads. The CPW line is composed of a signal line and two ground lines, and is used to transmit microwave signals. Its characteristic impedance is 50 Ω for the following measurement, where the width of the signal line and the distance between the signal and ground lines are designed to be 100 and 58 µm, respectively. In order to achieve the good matching relationship, the two load resistors with the resistance of 100 Ω are in parallel connected to one end of the CPW line. The length and the width of each resistor are 58 and 14.5 µm, respectively. The thermopile is placed close to the load resistors, and it is composed of twelve thermocouples that are connected in series to obtain large output thermovoltages. In the GaAs MMIC process, each thermocouple is made of n^+^ GaAs and AuGeNi/Au [17]. In the design, the distance between the resistors and the thermopile is 10 µm. When the microwave signal under test is input and transmitted to the CPW, the load matching resistors completely absorb the microwave power and generate heat based on the theory of Joule heat. The generated heat causes an increase in temperature around the resistors. So that, the temperature difference is formed at both ends of the thermopile. Finally, the resulting temperature difference is converted to the output thermovoltages by the thermopile, based on Seebeck effect. These power sensors do not require a dc bias during operation.

Therefore, the power sensors utilize the conversion principle of microwave power-heat-electricity. For a certain microwave power, the thermovoltage is related to the temperature difference. That is, the sensitivity of the power sensor is determined by the efficiency of heat transfer from the resistors to the thermopile. In order to increase the temperature at the end of the thermopile in proximity to the resistors, two methods are adopted. The one, the floating thermal slug is placed on the load resistors and the thermopile, and electrically isolated from the resistors and thermopile through using a Si_3_N_4_ dielectric layer. The thermal slug is made of gold. The dielectric layer is thin (0.23 µm) and it has little effect on heat conduction. So, the thermal slug can transfer the heat from the resistors to the thermopile. The method is equivalent to arranging a heat conduction path above the substrate, which increases the efficiency of the heat transfer. In this paper, the thermal slug is designed to be in the floating state, and differed from the grounded slug in [17]. It means that the floating thermal slug is not connected to the CPW ground line. The floating thermal slug, the Si_3_N_4_ layer and the load resistor layer constitute a MIM capacitor, and the capacitor will cause parasitic capacitive reactance. But, the effect of the capacitance on the resistance of the load resistors is small, to less than 5% from the simulation. So the advantage of such design is that the floating slug almost does not affect the resistance of the load resistors, thereby achieving good impedance matching. Thus, the floating design of the thermal slug is ability to obtain low reflection losses at a wider frequency rang. More importantly, it shows that more power is dissipated to generate heat, which contributes to increasing the sensitivity of the sensors. So, the floating thermal slug can achieve the low reflection loss at the wideband and improve the sensitivity. The other, the back cavity is etched by the MEMS technique, and the substrate membrane underneath the resistors and the hot end of the thermopile is formed. The thin substrate leads to a reduction in the heat losses of the substrate. The method is equivalent to arranging another heat conduction path in the substrate, which increases the efficiency of heat transfer. The thinner the substrate is, the smaller the heat losses of the substrate are. However, if a too thin membrane is etched, then the power sensors will bring challenges to the packaging and reliability. In order to ensure the packaging and reliability, the back cavity with a robust stiffness of the membrane is fabricated, where the substrate membrane is about 20 µm in thickness.

Based on the Seebeck effect, the output thermovoltages that are generated by the twelve thermocouples and can be expressed as:

(1) $$V_{out}=(\alpha_{1}-\alpha_{2})\sum_{i=1}^{i=N}\Delta T(i)$$

where α_1_ and α_2_ are Seebeck coefficients of n^+^ GaAs and AuGeNi/Au, *N* is the number of the thermocouples and equal to 7, and ∆*T*(*i*) is the temperature difference between the hot and cold ends of the named (*i*) thermocouple. According to the theory of the heat transfer equation, ∆*T*(*i*) can be written as [11]:

(2) $$\Delta T(x,y)=\sum_{n=1}^{+\infty }C_{n}(e^{\frac{n\pi }{W}x}-e^{\frac{n\pi }{W}(2L-x)})\sin\frac{n\pi }{W}y\begin{array}{cc} & n=1,\;2\;\cdots +\infty \end{array}$$

where *C_n_* is the coefficient that can be obtained, *L* and *W* are the length and width of the edge of the power sensors, respectively. Thus, the sensitivity of the thermoelectric power sensors is represented as:

(3) $$S=\frac{V_{out}}{P_{in}}$$

where *P_in_* is the input microwave power. Therefore, as seen in Equation (1), the output thermovoltages are proportional to the temperature difference between the hot and cold ends of the thermopile; as seen in Equation (2), the sensitivity of the power sensors is proportional to the thermovoltages.

**Figure 2:**
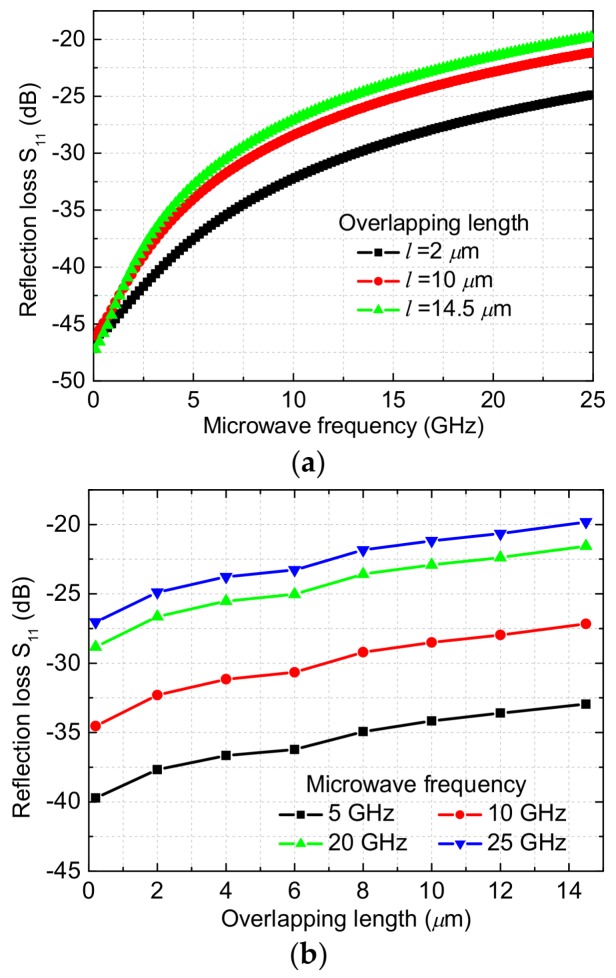
Simulated reflection losses of the GaAs MMIC-based power sensors. (**a**) S_11_ versus microwave frequency at the fixed overlapping length of 2, 10, and 14.5 µm and (**b**) S_11_ versus the overlapping lengths at the fixed frequencies of 5, 10, 20, and 25 GHz.

**Figure 3:**
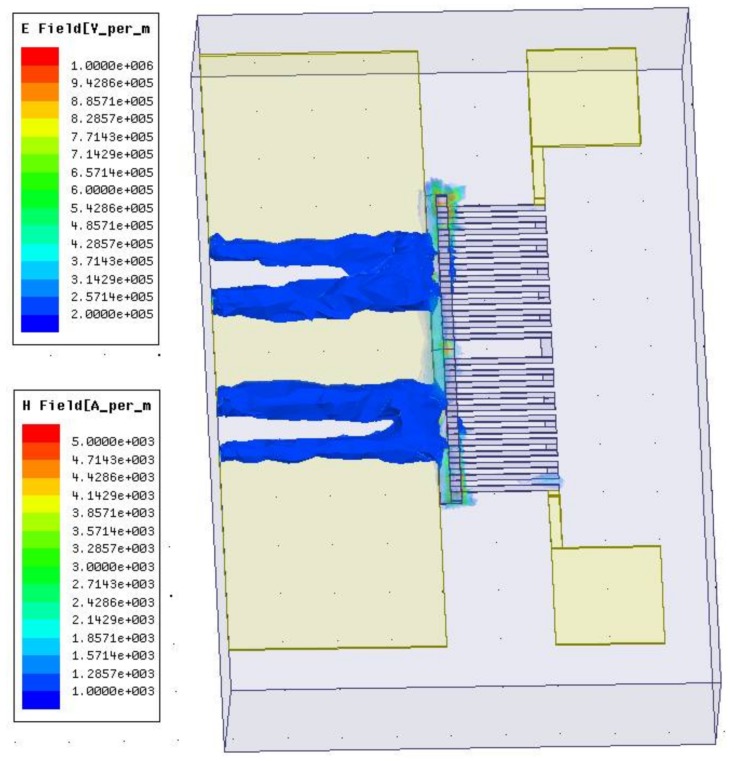
Simulated electromagnetic field distribution of the power sensor with the overlapping length of 10 µm between the floating thermal slug and the resistors.

In this paper, the MEMS thermoelectric power sensors with dual thermal flow paths are designed to operate up to 25 GHz. In order to analyze the effects of the floating thermal slug on the reflection losses, the power sensors are simulated and optimized by HFSS (High Frequency Structure Simulator). Figure 2a shows the simulated reflection losses of the GaAs MMIC-based power sensors with different overlapping lengths between the floating thermal slug and the load resistors. The simulated frequency range is from DC to 25 GHz. When the overlapping lengths between the floating thermal slug and the load resistors are 2, 10, and 14.5 µm, the corresponding percentages are 13.8%, 69.0%, and 100%, respectively. In general, the larger the overlapping length is, the higher the reflection loss at microwave frequencies is. This is because that the large overlapping length leads to the increase of the capacitance. For the overlapping lengths of 2, 10, and 14.5 µm, the simulated reflection losses are less than −24.8, −21.1, and −19.8 dB at DC-25 GHz, respectively. These results show that the proposed power sensors exhibit the low reflection losses, which verifies the design validity of the floating thermal slug. For the overlapping lengths of 2 and 10 µm, the optimized reflection losses are below −20 dB. Figure 2b shows simulated reflection losses versus the overlapping lengths between the floating thermal slug and the load resistors at the fixed frequencies of 5, 10, 20, and 25 GHz. At these frequencies, the reflection losses increase as the overlapping lengths increase. Figure 3 shows simulated electromagnetic field distribution of the power sensor for the overlapping length of 10 µm between the floating thermal slug and the resistors. As can be observed, a portion of the electromagnetic field is coupled to the thermopile through the floating thermal slug, but the amount is small. It means that there is a small effect on the sensing output of the thermopile.

**Figure 4:**
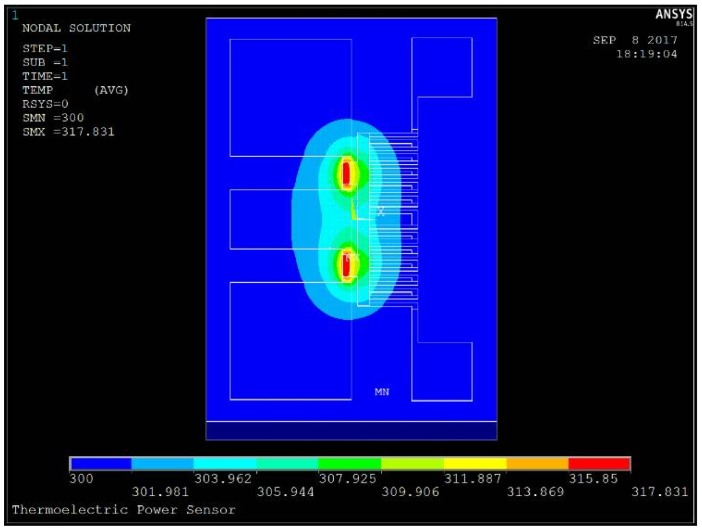
Simulated temperature distribution of the power sensor with the dual thermal flow paths under the power level of 100 mW.

**Figure 5:**
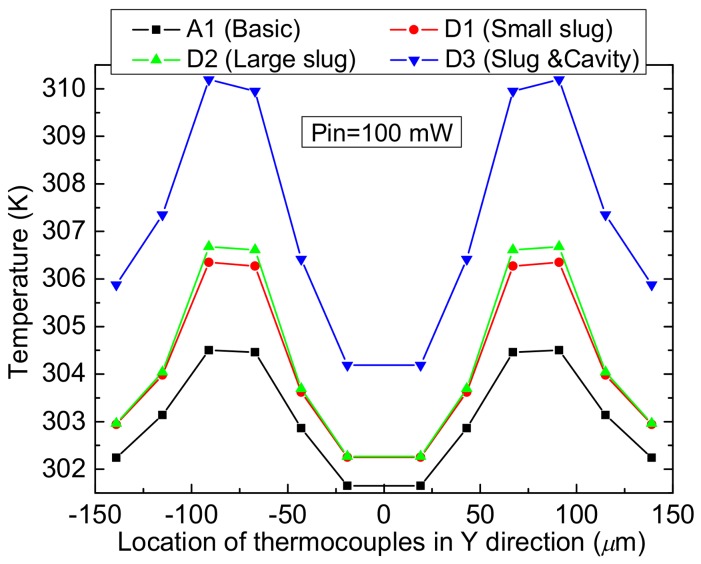
Temperature on hot junctions of the several thermocouples of the sensors A1, D1, D2, and D3 with respect to the location of the thermocouples in the Y direction when the power is 100 mW.

In order to show the heat transfer aspect, these power sensors are simulated by using an ANSYS software. Figure 4 shows the simulated temperature distribution of the power sensor with the dual thermal flow paths under the power level of 100 mW. In order to show the design validity, the basic and improved MEMS thermoelectric power sensors are given together. Here, their common sizes are same. The basic sensor is no thermal slug and cavity. The improved sensors D1 and D2 only have the floating thermal slug, where the overlapping lengths between the floating slug and the resistors are 2 and 10 µm, respectively. They are called as the sensors with small and large floating thermal slugs. The improved sensor D3 includes the floating thermal slug and the back cavity. It is called as the sensor with the dual thermal flow paths. Figure 5 shows the temperature on the hot junctions of the several thermocouples of the sensors A1, D1, D2, and D3 when the power is 100 mW. By comparing the three sensors A1, D1, and D2, they show that the thermal slug can act as the heat conduction path above the substrate and increase the efficiency of heat transfer. By comparing the four sensors, the sensor D3 with the floating thermal slug and the back cavity shows the highest temperature. Such results verify the design validity of the dual thermal flow paths.

## 3. Measurement and Discussion

In order to facilitate the performance comparison under the same process and experimental conditions, the basic and improved MEMS thermoelectric microwave power sensors are given together. These thermoelectric power sensors are fabricated using the GaAs MMIC process [17,18].

(i). The sensors start on a 3-inch GaAs wafer, and n^+^ GaAs is used to fabricate ohmic contact areas for the doping concentration of 1.0 × 10^18^ cm^−3^ and one leg of the thermopile for 1.0 × 10^17^ cm^−3^.
(ii). AuGeNi/Au is sputtered and patterned to form the other leg of the thermopile by a liftoff process.
(iii). TaN (square resistance of 25 Ω/□) is sputtered and patterned as the load resistors.
(iv). Ti/Pt/Au/Ti (500/300/3500/500 Å) is evaporated and patterned to form the CPW and pads.
(v). Si_3_N_4_ (1000 Å) is deposited by PECVD (Plasma Enhanced Chemical Vapor Deposition) and etched as the dielectric layer.
(vi). Ti/Au/Ti is evaporated as a seed layer, and Au (2 μm) is electroplated to thicken the CPW and pads and to fabricate the floating slug.
(vii). GaAs is thinned to 100 µm in thickness, and the substrate membrane underneath the resistors and the hot end is implemented by a via-hole etching technique.

**Figure 6:**
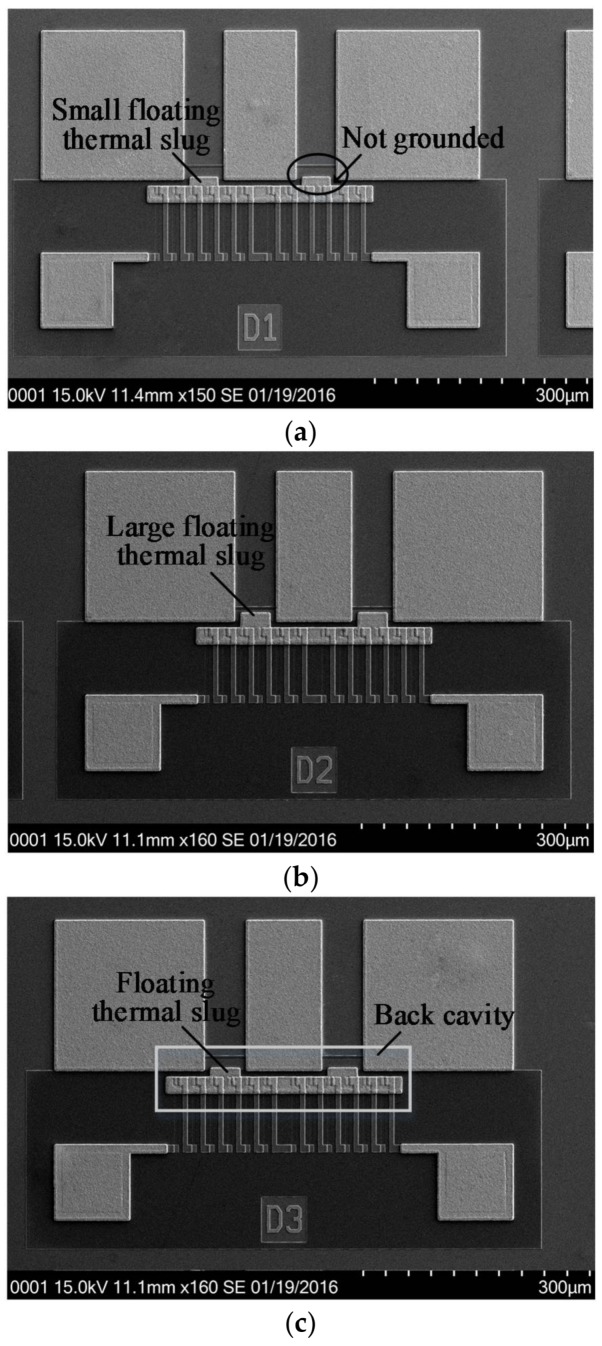
SEM views of three wideband and low-loss MEMS thermoelectric microwave power sensors in GaAs MMIC. (**a**) Sensor D1 with the overlapping length of 2 µm (small floating thermal slug); (**b**) Sensor D2 with the overlapping length of 10 µm (large floating thermal slug); and, (**c**) Sensor D3 with the floating thermal slug and the back cavity.

Figure 6 shows SEM (Scanning Electron Microscope) views of three wideband and low-loss thermopile-based microwave power sensors with the floating thermal slug and the back cavity in GaAs MMIC.

**Figure 7:**
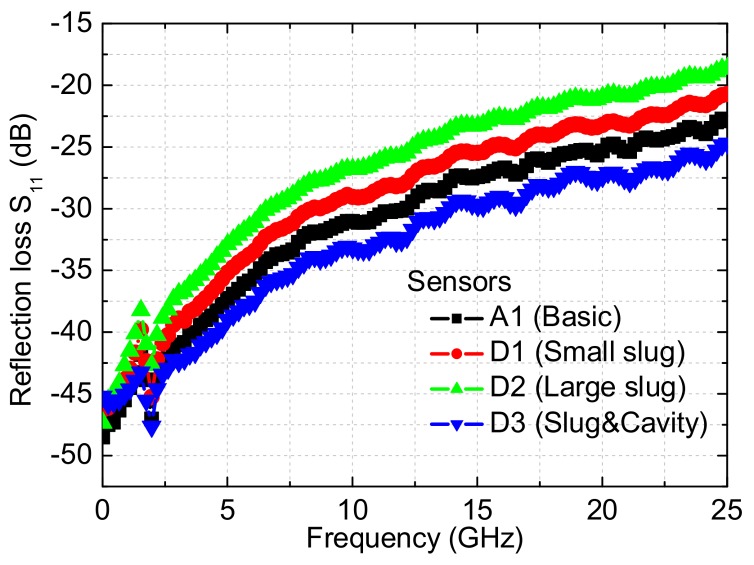
Measured reflection losses of the basic and improved thermopile-based microwave power sensors.

Microwave performances of the several power sensors are measured by the calibrated network analyzer and the probe station, and Figure 7 plots the results of the reflection losses. As for the basic sensor and the three improved power sensors, the measured reflection losses are less than −25.69 dB up to 12 GHz and −18.61 dB up to 25 GHz. It means that less than 0.28% (below 12 GHz) and 1.4% (below 25 GHz) microwave power is reflected back to the input port. So, they show the good impedance matching. For the basic sensor, the measured S_11_ are about −31.03, −25.33, and −22.90 dB at 10, 20, and 25 GHz, respectively. For the sensor D1 with the overlapping length of 2 µm, the measured S_11_ are about −29.05, −23.22, and −20.73 dB at 10, 20, and 25 GHz, respectively. For the sensor D2 with the overlapping length of 10 µm, the measured S_11_ are about −26.79, −20.96, and −18.62 dB at 10, 20, and 25 GHz, respectively. For the sensor D3 with the floating slug and the back cavity, the measured S_11_ are about −33.21, −27.41, and −24.73 dB at 10, 20, and 25 GHz, respectively. As can be observed in the sensors D1 and D2, the floating thermal slug with the overlapping length of 10 µm causes higher reflection losses than the slug with the overlapping length of 2 µm. However, when compared to the sensors with the grounded thermal slug in [17], they are smaller and acceptable for microwave applications. Such results verify the design validity of the floating thermal slug. In addition, the sensor D3 with the dual thermal flow paths exhibits the lowest reflection losses. The experiments demonstrate that the power sensors with the floating thermal slug can achieve low reflection losses in a wide frequency range. As for the thermopile-based power sensors, low reflection losses mean that less microwave power is reflected back and more power is dissipated to generate heat, which contributes to increasing the sensitivity of the sensors.

**Figure 8:**
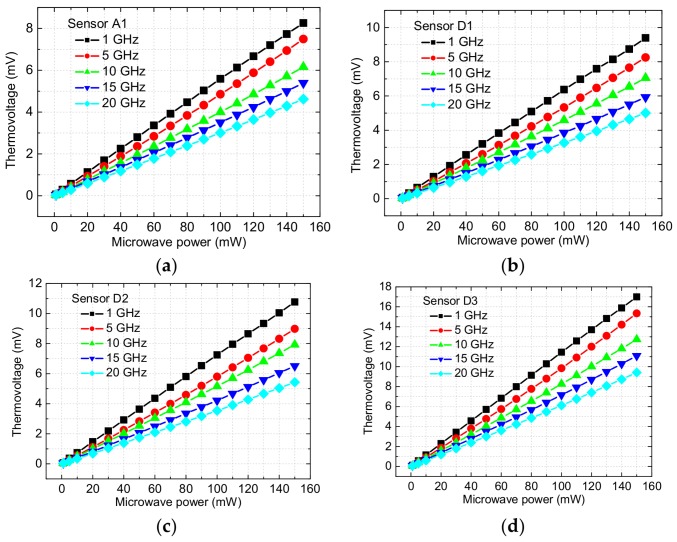
Measured thermovoltage as a function of the microwave power at 1, 5, 10, 15, and 20 GHz for the basic and improved MEMS power sensors. (**a**) Sensor A1 with the basic structure; (**b**) Sensor D1 with with the overlapping length of 2 µm; (**c**) Sensor D2 with the overlapping length of 10 µm; and, (**d**) Sensor D3 with the floating thermal slug and the back cavity.

When the microwave power is input through the CPW line, the thermovoltage is output and measured on the pads. Figure 8 shows the measured thermovoltages as a function of the microwave power at different frequencies for the basic and improved MEMS power sensors. In Figure 8, the thermovoltage increases as the microwave power changes from 1 to 150 mW at the fixed frequency, where the linear relationships between them are obtained. It means that the effects of the electrometric filed coupled by the floating thermal slug on the thermopile and its output are small. In other words, the power sensors can achieve a stable sensing output. Furthermore, the sequence of the thermovoltages is the sensor A1 < D1 < D2 < D3, at a fixed power and frequency. For example, when the microwave power is 150 mW for the sensors A1, D1, D2, and D3, the thermovoltages are about 8.26, 9.39, 10.77, and 17.00 mV at 1 GHz, 7.49, 8.25, 8.98, and 15.34 mV at 5 GHz, 6.16, 7.05, 7.93, and 12.73 mV at 10 GHz, 5.39, 5.93, 6.51, and 11.09 mV at 15 GHz, and 4.62, 5.01, 5.43, and 9.41 mV at 20 GHz, respectively.

**Figure 9:**
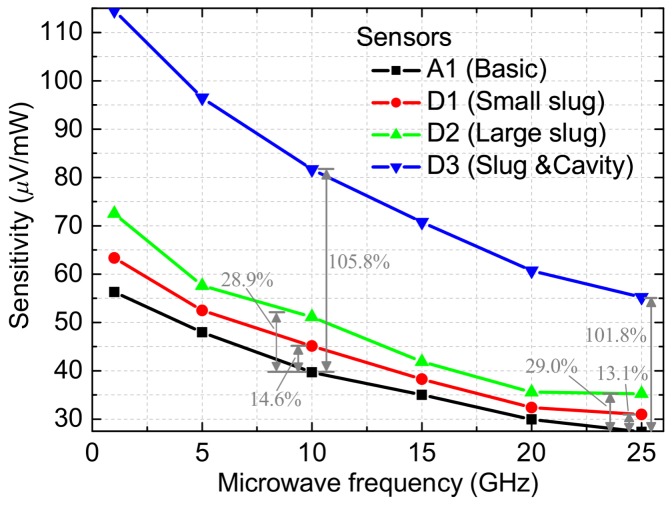
Average sensitivity as a function of the microwave frequency for the basic and improved MEMS power sensors.

Figure 9 shows the average sensitivity as a function of the microwave frequency for the basic and improved MEMS power sensors. At 1, 10, and 25 GHz, measured sensitivities are about 56.28, 39.68, and 27.36 μV/mW for the sensor A1 (basic), 63.33, 45.12, and 30.95 μV/mW for the sensor D1 (Overlapping length of 2 µm), 72.54, 51.13, and 35.28 μV/mW for the sensor D2 (Overlapping length of 10 µm), and 114.52, 81.68, and 55.20 μV/mW for the sensor D3 (floating slug and cavity). As can be observed in Figure 9, the sensors D1 and D2 have better sensitivities than the sensor A1. This shows the design validity of the floating thermal slug. Moreover, the sensitivities of the sensor D2 are higher than that of the sensor D1. It indicates that the large floating slug can better improve the heat transfer efficiency. At last, measurements show that the sensor D3 with the dual thermal flow paths (floating thermal slug and back cavity) produces the highest sensitivity. The results of these sensors are consistent with the design. When compared to the basic sensor A1, the proposed sensors D1, D2 and D3 in this paper generate improvements of 13.71%, 28.86%, and 105.85% at 10 GHz. The sensitivities of these relevant sensors with the floating slug are higher than those of the reported sensors with the grounded slug (e.g., 3.73% and 11.24% for the overlapping length of 2 and 10 µm (small and large grounded slugs), and 99.19% for the grounded slug and cavity at 10 GHz in [17]). This is because that the design of the floating slug in this paper leads to less reflection losses, which means that more power is dissipated to generate heat. This is equivalent to improving the sensitivity of the thermoelectric sensors.

**Figure 10:**
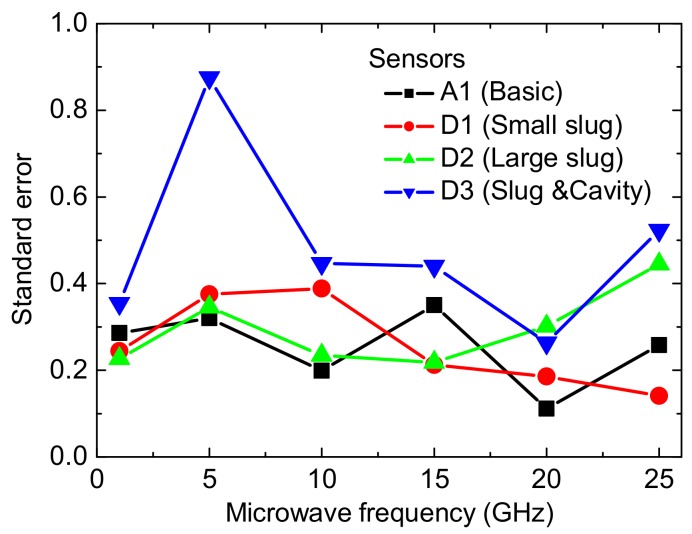
Standard error of the corresponding sensitivity in Figure 9.

Figure 10 shows the standard errors of the corresponding sensitivity in Figure 9. The error bars are small, which further shows the small effects of the electrometric filed on the output of the sensors. In other words, these sensors with the floating slug and the cavity can generate the stable output. In Figure 10, the errors in the sensitivity of the sensor D3 are a little higher than others. They result mainly from the air convection and the test environment. This is because all of the tests are performed at the room temperature and atmosphere condition, instead of a vacuum environment. For the thermopile-based sensors, a high temperature difference results in the large convective heat transfer, and the condition of the test environment affects the reference voltage of the multimeter (before the RF power is applied). Fortunately, as for the sensor D3, the errors relative to the sensitivity are small and acceptable. Table 1 shows the comparison of the MEMS thermoelectric power sensors in the GaAs process.

## 4. Conclusions

In this paper, the DC-25 GHz and low-loss MEMS thermoelectric power sensors with dual thermal flow paths are proposed for the GaAs MMIC and MEMS applications. In order to improve the sensitivity of the sensors, the collaboration usage of the floating thermal slug and the back cavity helps to increase the efficiency of heat transfer. The design of the floating slug achieves the low reflection of up to 25 GHz, accompanied by an equivalent improvement in sensitivity. The experiments demonstrate that these microwave power sensors produce low losses, wideband operation, and good sensitivity, with the enough stiffness membrane. The design method can be applied to similar thermopile-based devices.

## Figures and Tables

**Table 1:** Comparison of the MEMS thermoelectric power sensors in the GaAs process.

Ref.	Operation Frequency	Reflection Loss (dB)	Sensitivity (μV/mW)	Thickness of Substrate Membrane/Reliability	Membrane Process
[5]	dc-26.5 GHz	−29.4	16,400	1.5 μm/not good	No standard
[11]	dc-10 GHz	−26@10 GHz	160@10 GHz	10 μm/general	Standard
[17]	0.01–12 GHz	−23.15@12 GHz	79.04@10 GHz	20 μm/good	Standard
This work	dc-25 GHz	−32.61@12 GHz−24.73@25 GHz	81.68@10 GHz55.20@25 GHz	20 μm/good	Standard
